# Glutamate-Gated Chloride Channels of *Haemonchus contortus* Restore Drug Sensitivity to Ivermectin Resistant *Caenorhabditis elegans*

**DOI:** 10.1371/journal.pone.0022390

**Published:** 2011-07-26

**Authors:** Susan K. Glendinning, Steven D. Buckingham, David B. Sattelle, Susan Wonnacott, Adrian J. Wolstenholme

**Affiliations:** 1 Departmenty of Biology and Biochemistry, University of Bath, Bath, United Kingdom; 2 Wadham College, University of Oxford, Oxford, United Kingdom; 3 Faculty of Life Sciences, University of Manchester, Manchester, United Kingdom; 4 Department of Infectious Diseases, University of Georgia, Athens, Georgia, United States of America; 5 Center for Tropical and Emerging Global Diseases, University of Georgia, Athens, Georgia, United States of America; New England Biolabs, United States of America

## Abstract

Anthelmintic resistance is a major problem in livestock farming, especially of small ruminants, but our understanding of it has been limited by the difficulty in carrying out functional genetic studies on parasitic nematodes. An important nematode infecting sheep and goats is *Haemonchus contortus*; in many parts of the world this species is resistant to almost all the currently available drugs, including ivermectin. It is extremely polymorphic and to date it has proved impossible to relate any sequence polymorphisms to its ivermectin resistance status. Expression of candidate drug-resistance genes in *Caenorhabditis elegans* could provide a convenient means to study the effects of polymorphisms found in resistant parasites, but may be complicated by differences between the gene families of target and model organisms. We tested this using the glutamate-gated chloride channel (GluCl) gene family, which forms the ivermectin drug target and are candidate resistance genes. We expressed GluCl subunits from *C. elegans* and *H. contortus* in a highly resistant triple mutant *C. elegans* strain (DA1316) under the control of the *avr-14* promoter; expression of GFP behind this promoter recapitulated the pattern previously reported for *avr-14*. Expression of ivermectin-sensitive subunits from both species restored drug sensitivity to transgenic worms, though some quantitative differences were noted between lines. Expression of an ivermectin-insensitive subunit, Hco-GLC-2, had no effect on drug sensitivity. Expression of a previously uncharacterised parasite-specific subunit, Hco-GLC-6, caused the transgenic worms to become ivermectin sensitive, suggesting that this subunit also encodes a GluCl that responds to the drug. These results demonstrate that both orthologous and paralogous subunits from *C. elegans* and *H. contortus* are able to rescue the ivermectin sensitivity of mutant *C. elegans*, though some quantitative differences were observed between transgenic lines in some assays. *C. elegans* is a suitable system for studying parasitic nematode genes that may be involved in drug resistance.

## Introduction

Infections with parasitic nematodes are a major problem in animal health, and for human health in less-developed parts of the world [1]. Currently, the only effective means of control is by treatment with chemical anthelmintics [2]. One of the major classes of anthelmintic is the macrocyclic lactones (MLs), which include ivermectin and moxidectin, and these have been widely used in agriculture [3] and, in the case of ivermectin, human medicine [4], [5]. However, some species of parasite, especially those infecting small ruminants, have become resistant to the MLs, and this is threatening sustainable worm control in those animals [6], [7]. There have also been suggestions of the emergence of ML resistance in human parasites [8], [9], so there is an urgent need to understand the genetic basis of this resistance. Unfortunately, many informative genetic experiments in parasitic nematodes are extremely difficult, so for some time there has been an interest in exploiting the free-living species, *Caenorhabditis elegans*, for such studies, taking advantage of the powerful genetic and behavioural tools that can be applied to this organism[10]. *Haemonchus contortus* is an economically important parasite of small ruminants which has developed widespread resistance to anthelmintic drugs, including the MLs [6]. The major targets of the MLs are the glutamate-gated chloride channel (GluCl) receptors; activation or potentiation of these channels in GI nematodes by the drugs produces paralysis and inhibition of feeding [11], and in filarial nematodes inhibits protein secretion [12]. An improved understanding of the contribution of GluCl receptors to anthelmintic actions and resistance would aid in developing new pharmacological strategies against parasitic nematodes.

Investigation of the behavioural and drug target roles of individual GluCl subunits in parasitic nematodes is hampered by several limitations. The requirement for a host to complete the lifecycle of the parasite is a major obstacle in producing mutant strains of parasitic nematode required for a detailed analysis of GluCl subunit function. Transgenesis of parasitic nematodes is possible [13], [14], and some parasitic nematodes are more amenable to this strategy than others. For example *Strongyloides stercoralis* has been successfully transformed [15], [16], [17] although this parasite is unusual in that it has free-living generations which alternate with parasitic generations. *H. contortus* has not been successfully used in studies of transgenesis so far, although RNAi may be useful in the future for studying genes in *H. contortus* by gene knockdown [18], [19]. Due to the powerful genetic techniques available when working with the free living model organism *C. elegans*, our aim was to exploit *C. elegans* for the study of *H. contortus* GluCl subunit function *in vivo*. Successful expression of subunits from the parasite in *C. elegans* would enable us to interrogate the function of particular GluCl subunits.

Several other investigators have looked at the ability of parasite gene products to express successfully in *C. elegans*. Gillan et al., studying *hsp-90*, found that the *H. contortus* orthologue produced only a partial rescue of the *C. elegans* mutation, whereas the orthologue from *Brugia pahangi*, a more distantly related parasite, failed to produce any rescue [20]. Other *H. contortus* genes that have been successfully expressed in *C. elegans* include cathepsin L [21], the transcription factor *elt-2* [22] and β-tubulin, where Kwa et al [23] showed that polymorphisms associated with benzimidazole resistance would cause the transgenic *C. elegans* to become drug resistant. Genes from other parasites have also been expressed; Kampkotter et al [24] showed that a glutathione-S-transferase from *Onchocerca volvulus* would make *C. elegans* more resistant to oxidative stress and Massey et al. [25] showed that a forkhead transcription factor from *Strongyloides stercoralis* would rescue *daf-16* associated defects. In contrast, Crook et al. [26] found that a putative *daf-7* orthologue from *Parastrongyloides trichosuri* (*Ptr-daf-7* – genetic nomenclature as proposed by Beech et al. [27]) did not complement *C. elegans daf-7* mutants. Very recently, Welz et al [28] showed that expression of the *Ancylostoma caninum* SLO-1 channel could completely rescue the emodepside sensitivity of a *slo-1* null mutant of *C. elegans*, demonstrating the utility of the free-living nematode for the expression of parasite drug targets.

Studying genes that may be involved in macrocyclic lactone action and resistance in this manner has the potential to be challenging as there are multiple genes encoding ivermectin-sensitive GluCl subunits in nematodes. *C. elegans* has six GluCl genes, with at least two of these being alternatively spliced [29]; in *H. contortus* there are also six known GluCl genes coding for at least seven subunits, however the composition of the two gene families is not the same (Table 1). The gene family from *C. elegans* contains two genes, *Cel-glc-1* and *Cel-avr-15*, for which structural orthologues have not been described in *H. contortus* and the parasite has two genes, *Hco-glc-5* and *Hco-glc-6*, which are not present in *C. elegans* [27]. The subunits are, by analogy with nicotinic and GABA_A_ receptors, assumed to combine to form pentameric receptors, however the subunit composition and stoichiometry of the functional receptors *in vivo* in either species is unknown. Many, but not all, GluCl subunits form ivermectin-sensitive channels when expressed in vitro (Table 1). An important similarity between the two nematodes is the presence of the *avr-14* gene, which has structural orthologues not only in these two species, but in all nematode genomes examined so far [30], [31], [32]. This gene is alternatively spliced to produce AVR-14A and AVR-14B subunits; the alternatively spliced subunits share 81% and 82% amino acid sequence similarity between each other in *H. contortus* and *C. elegans* respectively [30]. The AVR-14B subunits have 87% amino acid sequence identity between the two worm species, while for AVR-14A the identity is 88%. The AVR-14 subunits share a common N-terminal ligand-binding domain but diverge thereafter in the sequence that forms the ion channel and intracellular domain. Previously we have shown that expression of Hco-AVR-14A and –AVR-14B in *avr-14* mutant *C. elegans* can rescue the behavioural abnormality, increased reversal frequency, associated with the mutation [33]. However, mutations in *avr-14* alone are not sufficient to make *C. elegans* resistant to ivermectin [34], so it was not possible to test the effects of the parasite subunits on drug resistance in those studies.

**Table 1:** The glutamate-gated chloride channel gene families of *C. elegans* and *H. contortus.*

*C. elegans*	*H. contortus*	Comments
*avr-14*	*avr-14*	Alternatively spliced to form two subunits [30], [31]. AVR-14B forms glutamate- and ivermectin-gated channels [34], [43].
*avr-15*		Alternatively spliced to form two subunits. Forms glutamate- and –ivermectin sensitive channels [48], [49].
*glc-1*		Forms ivermectin-sensitive channels when expressed alone; these are gated by glutamate when co-expressed with GLC-2 [36].
*glc-2*	*glc-2*	Does not form ivermectin-sensitive channels when expressed alone. Cel-GLC-2 forms glutamate-gated channels; these are sensitive to ivermectin when co-expressed with GLC-1 [36].
*glc-3*	*glc-3*	Cel-GLC-3 forms glutamate- and ivermectin-gated channels [50]. *Hco-glc-3* has only recently been identified.
*glc-4*	*glc-4*	No functional data. Sequence is somewhat divergent from other GluCl genes [51].
	*glc-5*	Forms ivermectin- and glutamate-gated chloride channels [52].
	*glc-6*	No functional data.

In the experiments reported here we make use of the triple GluCl mutant *C. elegans* line DA1316, which has 4,000 fold resistance to ivermectin in a growth assay compared to wild-type [34]. The three GluCl genes with mutations in DA1316 are *avr-14*, *avr-15* and *glc-1*, with the mutations in the former two genes producing null-function subunits for both splice variants. DA1316 was selected as the background mutant to test the hypothesis that GluCl subunits from *H. contortus* could rescue the ivermectin sensitivity of mutant *C. elegans*. In addition, we wished to examine whether other members of the GluCl gene family could also rescue an *avr-14* defect; whether only orthologous genes were capable of accurately rescuing the *C. elegans* phenotype or whether paralogous genes were also capable of restoring drug sensitivity. Overall, these experiments test the usefulness of *C. elegans* as an expression system for studying drug resistance and other genes from a parasitic species.

## Results

### *avr-14* Expression Pattern

**Figure 1:**
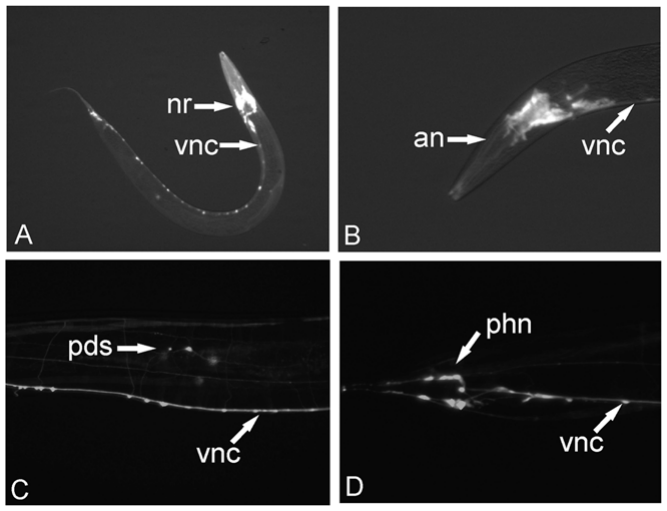
Expression pattern of GFP under the control of the *avr-14* promoter. The *avr-14* promoter fragment used was a section 1.7 kb upstream of the initiation codon. A) Whole adult wild type *C. elegans* transformed with a *Cel-avr-14::gfp* construct. vnc  =  ventral nerve cord; nr  =  nerve ring. B) Head section. an  =  amphidial neurones; vnc  =  ventral nerve cord. C) Body section between vulva and tail. pds  =  posterior deirid sensilla neurone cell bodies; vnc  =  ventral nerve cord. D) Tail section. phn  =  PHA, PHB and PHC neurone cell bodies. vnc  =  ventral nerve cord.

The expression pattern of wild type *C. elegans* transformed with a transcriptional reporter construct incorporating a 1,700 bp *avr-14* promoter fragment showed GFP expression in the nerve ring, ventral nerve cord and sensory neurones in the body (Fig. 1). This construct accurately reproduced the pattern shown previously using a similar *avr-14* promoter [34]. Expression was also clear in the amphidial neurones of the head and the phasmid neurones of the tail (Fig. 1).

### Rescue of ivermectin sensitivity of DA1316 with *C. elegans* and *H. contortus avr-14* cDNAs

**Figure 2:**
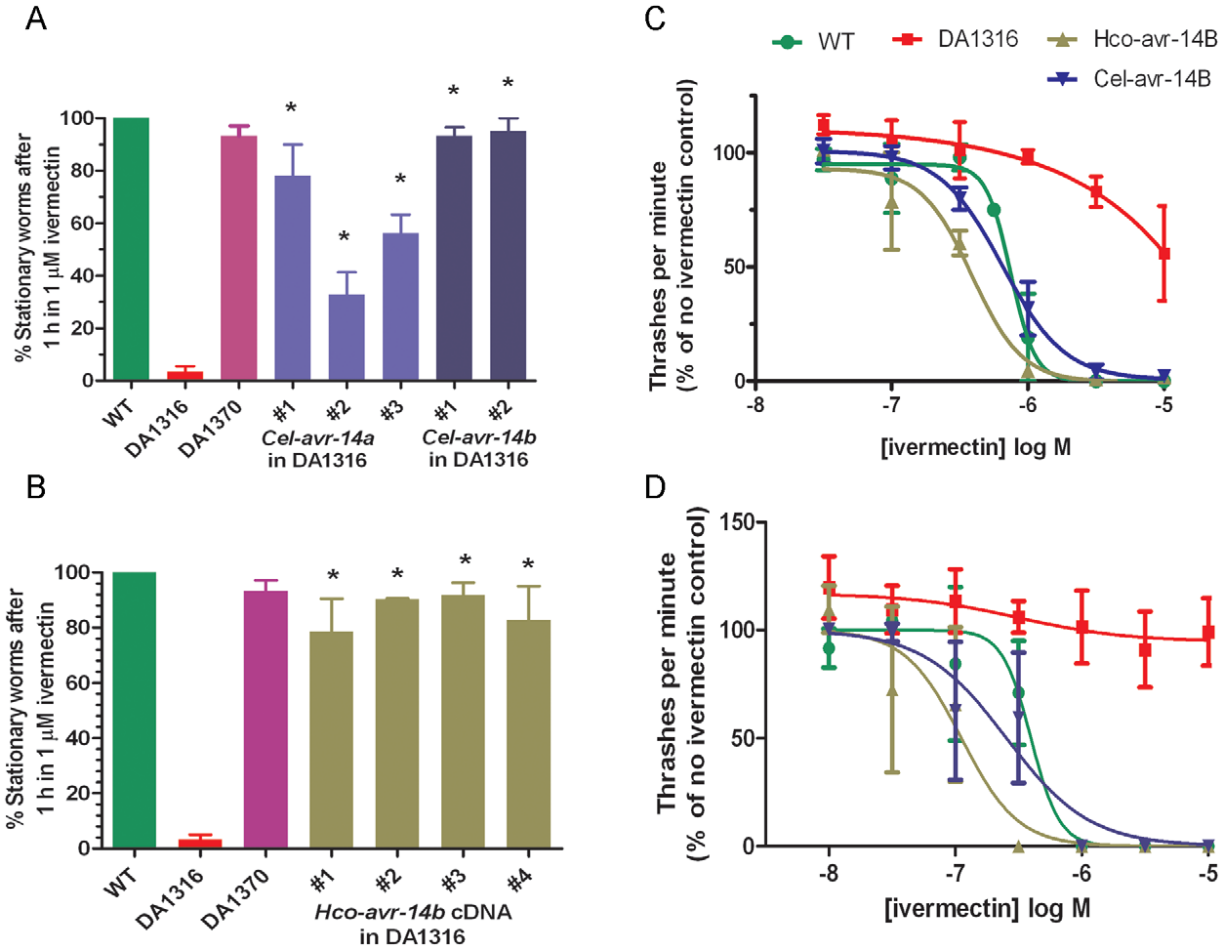
Motility assays showing rescue of ivermectin sensitivity by *avr-14* cDNAs in the resistant DA1316 mutant. N = 3. Asterisks indicate a significant difference (p = <0.05) between the transgenic lines and the parent DA1316 triple mutant (*avr-14; avr-15; glc-1*). A) Ivermectin sensitivity, assessed by counting the number of stationary worms after exposure to 1 µM ivermectin for 1 hr, of DA1316 transformed with either *Cel-avr-14a* (3 lines) or *Cel-avr-14b* (2 lines) cDNA. B) Ivermectin sensitivity of DA1316 transformed with *Hco-avr-14b* cDNA (4 lines), assessed as in panel A). For panels A & B, data are presented as mean ± SEM and a one-way ANOVA and Tukey's post-hoc test were performed on the two data sets separately using Minitab. Wild-type N2 worms and the DA1370 double mutant (*avr-15; glc-1*) are included for comparison. C) The effect of ivermectin on nematode swimming measured as “thrashes” per minute (as % of vehicle control) for wild type and transgenic *C. elegans* lines. *Hco-avr-14b* in DA1316 line 2 and *Cel-avr-14b* in DA1316 line 1 were chosen as representative lines from the motility assay for use in the thrashing assays. The experiment was repeated 3 times, with 8 worms per strain per concentration. D) An automated thrashing assay on the same lines as in panel C. The experiment was repeated between 1 and 4 times for each data point. Curves were fitted using the variable slope sigmoidal equation in GraphPad Prism (San Diego). The data are presented as mean ± SEM with N = 3. Symbols as in panel C).

The movement of the triple GluCl mutant strain, DA1316, was not affected by exposure to 1 µM ivermectin after 1 hour, in contrast to the total paralysis of 100% of wild type *C. elegans* that was observed under the same conditions (Fig. 2). In order to confirm that this resistance could be reversed by expression of one of the genes mutated in DA1316, *avr-14*, we transformed DA1316 with three subunit cDNAs (*Cel-avr-14a*, *Cel-avr-14b* or *Hco-avr-14b*) under the control of the *avr-14* promoter. Transformation with any of these cDNAs resulted in rescue of ivermectin sensitivity to varying degrees (Fig. 2A and B), with statistically significantly greater numbers of worms paralysed by exposure to 1 µM ivermectin in all the transgenic worm lines tested, compared to the parent DA1316. In particular, the *avr-14b* cDNA of both nematode species resulted in a robust rescue, with at least 79% of the tested worms becoming immotile in the presence of ivermectin. This result was obtained with two different lines expressing the *C. elegans* subunit, where 93±3.4% and 95±5.0% of the worms were paralysed, and four lines expressing the *H. contortus* subunit, where the proportion that were paralysed varied from 79±12.1% to 92±4.5%. The results with the lines transformed with both the *C. elegans* and *H. contortus avr-14b* cDNAs were similar to both wild-type worms and to DA1370, a double mutant strain (*avr-15; glc-1*) that is only weakly resistant to ivermectin [34]. The three lines containing *Cel-avr-14a* cDNA in DA1316 showed different levels of ivermectin sensitivity, from 33±8.6% of line 2 paralysed to 78±11.8% of line 1; the differences between *avr-14A* lines 2 & 3, and the *avr-14B* lines were significant (p = <0.01).

In an attempt to obtain more quantitative data on the level of drug sensitivity of the transgenic lines, we carried out thrashing assays in varying concentrations of ivermectin, and compared the results obtained by counting manually with those obtained with an automated system [35]. In summary, the thrashing rates of the transformed worms were similar to those of wild type, showing a greater sensitivity to ivermectin than the untransformed triple mutant, DA1316. There was no significant difference between the thrashing rates (75±16 to 95±4 thrashes/min) of any of the lines when placed in 0.1% (v/v) DMSO as a vehicle only control. The dose-response curve of DA1316 indicated that these worms become susceptible to the paralytic effects of ivermectin at concentrations above 10 µM (Fig. 2C). Even at 10 µM ivermectin, *C. elegans* with mutations in the three GluCl subunit genes reduced their thrashing rate by only 50%. In contrast, the transformation of DA1316 with *avr-14b* cDNA from either *C. elegans* or *H. contortus* resulted in an increase in the sensitivity of these worms to ivermectin to such an extent that they had a thrashing rate of only 2±1 and 2±2 thrashes/min respectively at 10 µM drug, and their thrashing rate was reduced by more than 50% at 1 µM ivermectin. The results from transgenic worms expressing Cel-AVR-14A were more variable, with two out of three lines retaining some activity in 1 µM ivermectin (Fig 2A).

The automated thrashing assay produced very similar results to those obtained by the more laborious manual thrashing assay, with rescue of the ivermectin sensitivity observed for DA1316 expressing all of the avr-14 cDNAs (Fig. 2D). The nature of the assay allowed us to measure more ivermectin concentrations, and also more *C. elegans* strains simultaneously than was possible with the manual thrashing assay. Importantly, very similar thrashing rates and dose-response curves were produced using the two different methods.

### Chronic Ivermectin Sensitivity Assay

**Figure 3:**
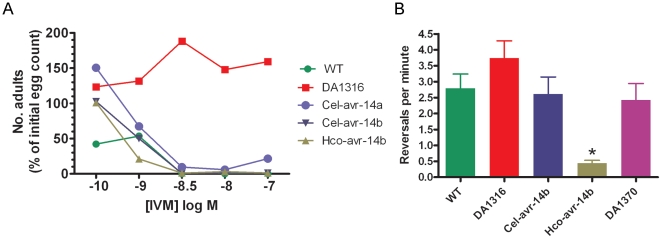
Development and behavioural assays demonstrating rescue of the *avr-14* phenotypes. A). The number of eggs that developed to adulthood on different concentrations of ivermectin. There is rescue of ivermectin sensitivity by *Cel-avr-14a*, *Cel-avr-14b* and *Hco-avr-14b* cDNA in the resistant DA1316 mutant. Data are presented as mean ± SEM. B) The DA1316 triple mutant transformed with *Hco-avr-14b* cDNA carried out fewer reversals per minute than the other lines. Reversals per minute were counted to compare WT, DA1316 (*glc-1*; *avr-14*; *avr-15*) and DA1316 containing either *Cel-avr-14b* or *Hco-avr-14b* cDNA behind the *Cel-avr-14* promoter. The double mutant, DA1370 (*avr-15; glc-1*), which has mutations in *glc-1* and *avr-15* was also used as a comparison. * Indicates that the reversals of the line containing the cDNA from the parasite were significantly reduced compared to DA1316, WT and DA1316 containing *Cel-avr-14b* cDNA. No difference was detected between the reversal frequencies of WT, DA1316, DA1370 or *Cel-avr-14b* in DA1316, using a one-way ANOVA (Minitab). Data are presented as mean ± SEM with N = 10 worms for each line.

The previous assays both measured the sensitivity of the worms to an acute exposure to ivermectin. However *in vivo* drug exposure would be much longer-term, with potential effects at lower concentrations than were used in the motility assays. The chronic ivermectin sensitivity assay, or growth assay, involved placing eggs onto nematode growth medium containing different concentrations of ivermectin and is a useful measure of the combined effects of ivermectin on foraging (locomotion) and pharyngeal pumping activity in different lines of *C. elegans*. Control plates containing 0.1% (v/v) DMSO were also set up during each experiment to account for any non-ivermectin related effects. The triple mutant line was resistant to ivermectin throughout the concentration range used, whereas no wild type adults were observed at concentrations above 1 nM ivermectin (Fig 3A). All the *avr-14* cDNAs conferred ivermectin sensitivity onto DA1316, and no adults were observed at concentrations of 3nM and above.

### Behavioural Assay

Our previous study had indicated that mutations in GluCl subunits affected the reversal frequency of *C. elegans*, with *avr-14* mutants reversing more frequently than wild-type, and also that expression of the *H. contortus* AVR-14B could rescue this abnormality in an *avr-14* mutant strain [33]; indeed, expression of *H. contortus* AVR-14B caused the worms to reverse less frequently than wild-type. However, the experiments in that study had not included DA1316, which has an additional mutation in *glc-1* [34], so we tested whether DA1316 also reversed more frequently, and to identify whether any altered number of reversals could again be rescued using *Hco-avr-14b* or *Cel-avr-14b* cDNA (Fig. 3B). The reversal frequency of DA1316 was not significantly elevated compared with wild-type, indicating that loss of a functional *glc-1* offset the increase caused by mutations in *avr-14* and *avr-15* [33]. DA1370, the double mutant (*avr-15;glc-1*) also reversed at the same frequency as the wild-type worms, adding additional support for this suggestion. The expression of Cel-AVR-14B had no effect on the reversal frequency of DA1316, but transforming the triple mutant with *Hco-avr-14b* cDNA produced worms with a severely reduced ability to reverse at normal frequencies.

### Rescue by other *C. elegans* and *H. contortus* GluCl subunits

**Figure 4:**
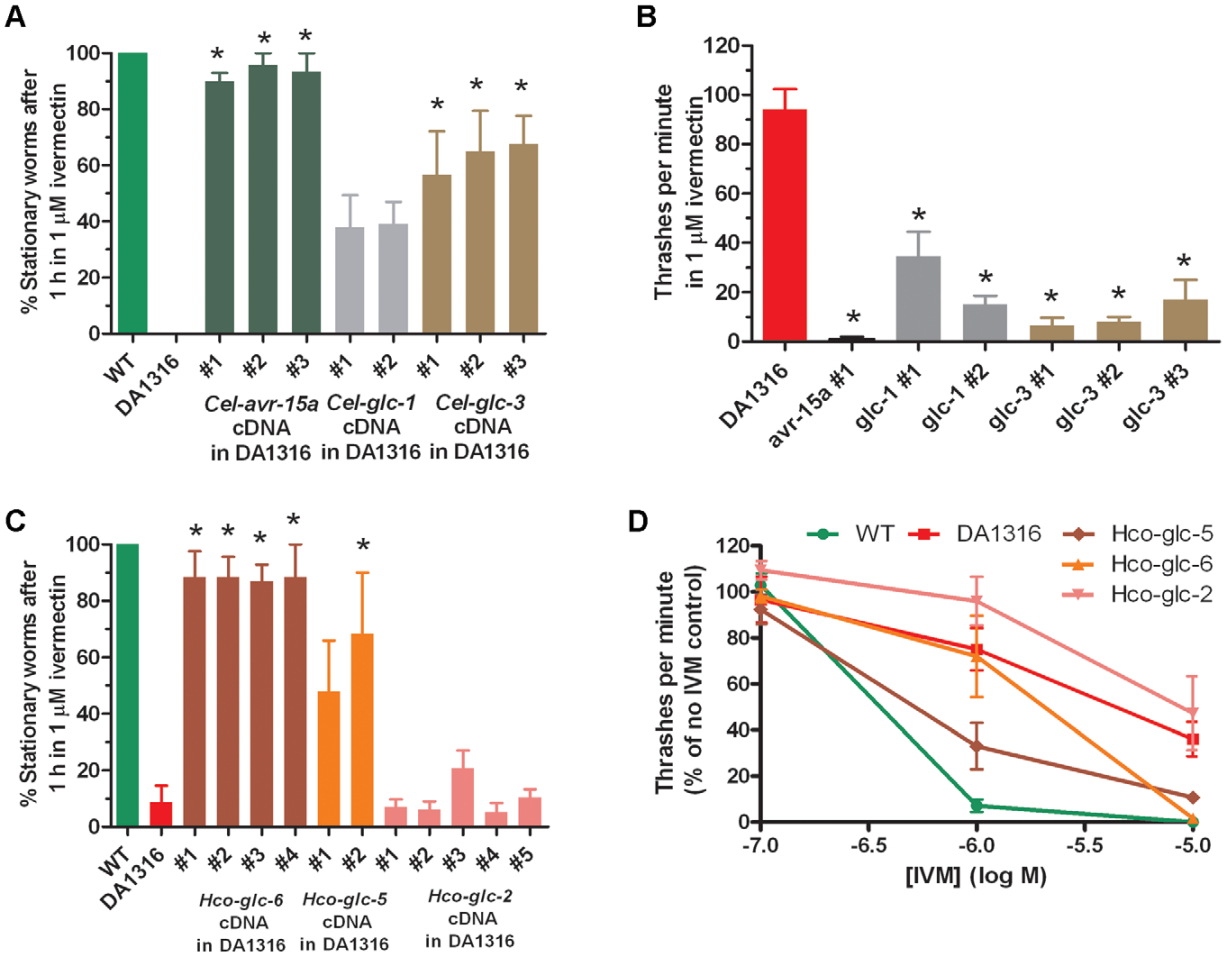
Other ivermectin-sensitive subunits from both *C. elegans* and *H. contortus* rescue the drug resistance of DA1316. A) Paralysis assay on lines transformed with cDNA expressing other *C. elegans* GluCl subunits. The proportion of worms that were paralysed by immersion in 1 µM ivermectin for 1 hr is shown. B) Thrashing assay of the lines from panel A. The number of thrashes/minute of the worms following a 1 hr exposure to 1 µM ivermectin is indicated. C) Paralysis assay on worms transformed with cDNAs expressing *H. contortus* GluCl subunits. D) Thrashing assay at three different concentrations of ivermectin, carried out on some of the lines shown in C). For panels A, B & C, *  =  statistically significant difference from DA1316 (p≤0.05).

The GluCl are pentameric channels, there are multiple GluCl subunit genes in both *C. elegans* and *H. contortus* and the subunit composition of the native channels is not known. *In vitro*, Cel-GLC-1 and -GLC-2 can co-assemble to form functional channels [36]; *in vivo*, *Cel-glc-2* is expressed only in the pharynx [37], where *Cel-avr-15* is also expressed [38], and the expression pattern of *Cel-glc-1* has not yet been determined. In *H. contortus,* the expression patterns of Hco-AVR-14 and Hco-GLC-5 overlap [39], though whether the two subunits co-assemble is not known. If, *in vivo*, AVR-14 subunits co-assemble with others to form ivermectin-sensitive heteromeric channels, this might mean that expression of non-orthologous GluCl genes would not rescue the ivermectin-resistance phenotype effectively, perhaps limiting the usefulness of this approach for studying genes from other species. We therefore transfected DA1316 with cDNAs encoding other ivermectin-sensitive subunits from *C. elegans* (*glc-1, glc-3, avr-15*) and *H. contortus* (*Hco-glc-5*). We also used cDNA encoding an ivermectin-insensitive subunit, Hco-GLC-2 [40] and an uncharacterised subunit, Hco-GLC-6 [27], from *H. contortus*. All of the cDNAs were sub-cloned behind the same *avr-14* promoter used previously. The results, shown in Figure 4, show that of the *C. elegans* subunits, all three lines expressing Cel-AVR-15 were almost completely sensitive to 1 µM ivermectin, whereas those expressing Cel-GLC-3 or Cel-GLC-1 were only partially sensitive to this concentration of the drug. Expression of the *H. contortus* GLC-5 subunit also caused a partial rescue of the sensitivity to 1 µM ivermectin, and complete sensitivity to 10 µM, whereas expression of Hco-GLC-2 did not produce any significant changes to DA1316, which is consistent with the insensitivity of GLC-2 channels to ivermectin [36], [40]. The uncharacterised Hco-GLC-6 subunit produced an almost complete rescue of drug sensitivity, indicating that it either produces ivermectin-sensitive channels, or can combine with pre-existing subunits to form such channels.

## Discussion

The study of polymorphisms and mutations potentially involved in anthelmintic resistance of parasitic nematodes is complicated by the difficulty of carrying genetic experiments on these organisms. Though *in vitro* studies are useful, especially in the case of those drugs which affect ion channels, *in vivo* confirmation that a particular sequence change does alter the organism's response to drug application is always desirable. *C. elegans* has been used as an expression system for many parasite genes, including those involved in resistance to the benzimidazole anthelmintics [23], and so it seemed an obvious question to ask if it is a suitable system for studying genes which encode targets of the MLs, and are thus candidate resistance genes [41]. The MLs act on glutamate-gated channels, either directly activating them or potentiating their response to glutamate [11]. The GluCls are expressed widely in the nematode nervous system, and are encoded by a small gene family; this family differs in size and composition between species, with some genes, such as *avr-14*, being widely conserved and others seeming to be present in much fewer species [27], [29], [32]. The presence of multiple potential targets in parasitic species, some of which do not have orthologues in *C. elegans*, as is the case for *H. contortus*, raises some interesting problems, especially if they are required to assemble with other subunits to form heteromeric receptors in order to be fully functional.

We addressed some of these issues by expressing multiple GluCl subunits from both *C. elegans* and *H. contortus* in a *C. elegans* strain (DA1316) that is very resistant to ivermectin, and by testing the sensitivity of the transgenic worms to the drug. DA1316 is a triple mutant strain (*avr-14, avr-15, glc-1*) but the double mutant (*avr-15, glc-1*) has a very low-level of resistance [34], so that even partial rescue of the *avr-14* mutation would produce a clear shift in response to the drug. A rescue of ivermectin sensitivity implies that the recombinant subunit forms, either by itself or by assembling with other endogenous polypeptides, a functional drug-sensitive channel. We chose *H. contortus* as the source of the parasite cDNAs for this study because it is a member of clade V like *C. elegans*, and is therefore not too phylogenetically distant [42], and because it encodes a number of ivermectin-sensitive GluCl subunits, not all of which have orthologues in the *C. elegans* genome (Table 1). The expression of GFP under the control of the *avr-14* promoter fragment showed that any recombinant subunits would be expressed in the same cells as the *C. elegans avr-14* gene products and, as predicted by our previous results [33], expression of both Cel-AVR-14B and Hco-AVR-14B restored the worms to almost complete drug sensitivity. A caveat to that statement is that we do not know which of the splice variants, AVR-14A or AVR-14B, are normally expressed in these cells, nor should it be assumed that the expression of this gene in parasitic species would be the same as in *C. elegans.* Indeed, for both *H. contortus* and *Brugia malayi*, there is evidence that it is not [12], [39]. Despite this, expression of Hco-AVR-14B not only rescued a behavioural defect associated with *avr-14*, an increased reversal frequency, but caused the worms to reverse less frequently than wild-type, as previously reported [33]. This unexpected result may be related to the much greater potency of L-glutamate on the Hco-AVR-14B receptor (EC_50_ = 27.6 µM) than on Cel-AVR-14B (EC_50_ = 2.2 mM) that we observed *in vitro* [43]. If one of the roles of AVR-14 is to suppress the initiation of reversals [33], then activation of Hco-AVR-14B by lower concentrations of glutamate may cause this suppression to last longer than in wild-type worms. The observation that DA1316 reversed with a similar frequency to wild-type is presumably due to the mutation in *glc-1*, which must be able to suppress the increase in reversal frequency caused by the *avr-14* and *avr-15* mutations. We have previously shown that not all mutation in GluCl genes cause an increase in reversal frequency; a deletion in *glc-3* causes a marked decrease in reversal frequency [33]. This is supported by the result with DA1370, which carries mutations in *avr-15* and *glc-1*. This strain showed no abnormality in reversal frequency (Figure 3B), unlike *avr-15* single mutants [33] which strongly suggests that the *glc-1* allele is suppressing this effect of the *avr-15* mutation The ivermectin resistance of the transgenic lines was measured in a variety of assays, and all gave essentially the same result, that the resistance of DA1316, whether to acute or chronic exposure, was reversed by expression of the AVR-14 subunits (Figures 2 & 3).

Mutations in the GluCl of ivermectin-resistant isolates of parasitic nematodes have been observed, in particular in *Cooperia oncophora* [44], and these mutations affect the sensitivity of the channels to ivermectin *in vitro* [43], [44]. In addition, mutations in the have been made in both the mammalian glycine receptor and the nematode GluCl that reduce or abolish the effect of ivermectin [45]. These amino-acid residues are found in M3 and contribute to the recently described binding site of ivermectin in the membrane domain of the GluCl [46]. It would be interesting to test such mutants of AVR-14B in transgenic *C. elegans*, to confirm that they do not rescue drug sensitivity and to examine whether the decreased reversal frequency shown in Figure 3B is still observed, and correlates with the *in vitro* potency of glutamate at these receptors.

These experiments showed that orthologous gene products from a parasitic nematode were functionally expressed in *C. elegans*, so we then looked at some paralogous gene products from both species. Figure 4 shows that those subunits that formed ivermectin-sensitive subunits *in vitro* also rescued the drug resistance phenotype, at least to some extent, though there were some quantitative and qualitative differences between some of the lines expressing paralogues and orthologues. Such a rescue could have come about in one of two ways; either the paralogous subunits were able to assemble with the normal partners of the endogenous AVR-14 subunits to form a functional GluCl, or they formed novel ivermectin-sensitive channels that silenced *avr-14* expressing neurones on exposure to the drug. The ivermectin insensitive subunit, Hco-GLC-2 [40], was not able to rescue ivermectin sensitivity but interestingly Hco-GLC-6 [27], which has not yet been studied by *in vitro* expression in *Xenopus* oocytes or in cell culture, was able to rescue the *avr-14* defect, suggesting that this subunit also contributes to an ivermectin-sensitive channel.

Taken together, the data show that *C. elegans* is a good system for the expression of parasite ion channels that act as drug targets, and is likely to be a reliable way of assessing the contributions of sequence polymorphisms within these subunits to a drug resistance phenotype. If this is true for ivermectin and the GluCl, then similar assays should be useful for the many drugs that act at nicotinic acetylcholine receptors, especially those that cause paralysis. The use of the model organism may help to overcome the formidable barriers to carrying out genetic experimentation on parasites.

## Methods

*C. elegans* wild-type and mutant strains were cultured according to standard techniques, and were maintained at 19°C on nematode growth medium (NGM) agar plates seeded with OP50 bacteria.

### Transformation of *C. elegans*

The *C. elegans* and *H. contortus* GluCl cDNA fragments were cloned behind a *Cel-avr-14* promoter fragment, as described previously [33]. The *avr-14* promoter was a 1700 bp fragment from immediately upstream of the *avr-14* translational start site. To test the expression pattern of this promoter fragment, a transcriptional GFP reporter construct was made by cloning the *avr-14* promoter fragment into a vector containing GFP (pPD95.75 Fire Vector Kit 1995, Addgene plasmid 1494) [47]. The reporter construct was used to transform N2 (wild type) *C. elegans* and the expression pattern examined (MZ16 F Fluorescence Stereomicroscope, Leica) (Fig. 1). The sequence of the Hco-glc-6 cDNA is deposited under Accession number EU006789.

Transformation of *C. elegans* was carried out using DNA-coated gold particle bombardment (Bio-Rad PDS-1000 with hepta adaptor for increased transformation efficiency). For the rescue experiments, the DA1316 strain (*avr-14*; *avr-15*; *glc-1* triple mutant: [34]) was bombarded using a total of approximately 3 µg of DNA. The co-transformation marker (*myo-3*::*gfp*, pPD118.20 Fire Lab Vector Kit 1997, Addgene plasmid 1592) and the test DNA were on separate plasmids, which were linearized by digestion with *Apa*I or *Kpn*I in standard buffers (NEB) in a total reaction volume of 50 µl for each plasmid. The DNA solutions were then mixed together and precipitated onto gold particles. Transformation was carried out under 1500 psi of helium gas pressure and a chamber vacuum of 21 mmHg. Subsequently, transformants expressing GFP in the body wall muscle were picked and successful lines were maintained. The presence of the test DNA was verified for each line using single worm PCR, with primers either specific to the start and end of the cDNA sequence (for *H. contortus* cDNA) or by designing primers that spanned exon-exon junctions (for *C*. *elegans* cDNA). In practise, 100% of lines expressing the *myo-3::gfp* construct contained the test DNA. At least two separate lines were produced for each GluCl cDNA tested. A control line was produced by transforming DA1316 with the *myo-3::gfp* construct only, and this line is used in place of untransformed DA1316 throughout the rest of the assays.

### Acute ivermectin sensitivity assays

#### Motility assay

A survey of the ivermectin sensitivity of the multiple lines was carried out for all worm strains produced. Ten individual worms were picked from each line/strain into 96-well plates with wells containing 50 µl M9 salts, two wells per line. 50 µl of 2 µM ivermectin solution was added to each well to produce a final concentration of 1 µM. The worms were left at 19°C for 1 h, after which time the number of stationary worms in each well was counted. The experiments were carried out in triplicate and using the data from this assay, one representative line containing each construct was chosen for use in the thrashing assay, chronic ivermectin sensitivity assay and behavioural assay.

#### Thrashing assay

Thrashing assays were carried out at different concentrations of ivermectin to characterise the drug sensitivity of the *avr-14B* transgenic lines more fully. The thrashing assay was carried out using two different methods: manual thrashing assay and automated thrashing assay. The two methods were used to verify the measurements thoroughly.

For the manual thrashing assay, individual young adult *C. elegans* were placed in 100 µl of ivermectin solution (made up in M9 buffer) in a 96-well microtitre plate. After 30 minutes in the drug at 19°C, the number of thrashes (bends of the body from one side to the other and back) were counted for 1 minute. A preliminary time-course investigation (data not shown) indicated that 30 minutes would be the appropriate duration in the drug at the ivermectin concentrations used (0.03 to 8.75 µg/ml). The experiment was carried out for 8 worms per strain, per ivermectin concentration and was repeated on two further days. The GluCl-transformed lines were chosen as representative lines, after inspection of the motility assay data. One thrash was counted as a movement of the head to one side *e.g.* a thrash was counted every time the head of the worm moved to the left.

The automated thrashing assay was carried out as for manual thrashing, except that the thrashing rates for all worms could be measured simultaneously. Movies were taken of the whole 96-well plate, and thrashing rates calculated for each well by the co-variance method as described previously [35].

### Chronic ivermectin sensitivity assay

NGM was made, allowed to cool, and spiked to contain a final concentration of either 0.01% DMSO or ivermectin at 100 pM or 1, 3.2, 10, or 100 nM. The NGM was transferred to 12-well plates, allowed to set and seeded with 50 µl OP50. After 2 days at room temperature, egg preparations of the different *C. elegans* lines were made, and eggs were transferred onto the ivermectin/DMSO plates. The number of adult worms in each well was counted six days later. Three replicate wells per ivermectin/DMSO concentration per worm strain were set-up, and the whole experiment was repeated on two subsequent occasions.

### Behavioural assay – reversal frequency

The frequency of reversals was as described by Cook et al [33]. Individual worms were picked onto an unseeded NGM plate (to remove traces of bacteria), and then transferred to the measurement NGM plate (also unseeded) where they were left to acclimatise for 5 min. The number of reversals (initiations of backward movement) in 5 min was counted.
